# The Book World of Medicine and Science

**Published:** 1895-04-06

**Authors:** 


### THE BOOK WORLD OF MEDICINE AND SCIENCE.

The Medical Annual. (Bristol: John Wright and Co. 1895.)

The editors of this annual have succeeded in compiling a first class retrospect of the last twelve months' progress in medical and surgical knowledge. The article on diphtheria, dealing as it does with the most engrossing subject of the year, will probably be read with interest. It has been carefully written by Dr. Armand Ruffer, and although not entirely free from optimism is on the whole a very fair and straightforward statement of facts, and expresses the views most generally accepted by the medical profession. Sanitary science and practical sanitation have been by no means neglected, and Dr. Priestley, of Leicester, has managed, within the narrow limits of twenty pages, to include most of the important improvements in this branch of medcine. He pays special attention to the subject of interceptor's traps, disinfectants and methods of disinfection. New surgical and medical appliances receive their full share of attention, and the pages devoted to this subject, are of peculiar interest to the practitioner who desires to provide his patients with all the latest improvements in sick-room accessories. An interesting article from the pen of Mr. Simeon Snell deals with the subject of eyesight and school life. The author draws attention to the desks and seating of scholars, and to quote from his article, "This matter of seating children is one of the most important, if we are to prevent short sight and crooked backs." Slanting *versus* upright handwriting, hours of study, and the lighting of schools are all important factors in school hygiene, and are ably dealt with by the same contributor.

The Droitwich Brine Baths. By W. H. Tomlins. (London: H. K. Lewis. Price 1s.)

In this pamphlet the author epitomises the therapeutic value of brine baths in general, and those of Droitwich in particular. His information is drawn chiefly from Von Ziemssens' "Handbook of General Therapeutics," and from Dr. Roden's work, "Droitwich and its Baths." We have utterly failed to find any original observation or suggestion, and it is difficult to discover the reason for its appearance, unless it be that the author, who has lived some part of his life in New South Wales, is infused with a spirit of patriotism on his return to his own country. Thus, at the close of the pamphlet, we notice the following sentence, "And the patriotic medical man who sends his patients here instead of to some foreign spa will have the satisfaction of knowing . . . that he is giving them quite as great, if not a greater, chance of recovery than they would have elsewhere." The patient intending to visit Droitwich, or the medical man seeking a suitable spa for rheumatic or gouty subjects, will find most of the information he can want in Mr. Tomlins' leaflet. Mr. Tomlins does not mention that there is at Droitwich an excellent cottage hospital with something like 60 beds, where a poorer class of patients is taken free of charge. The record of this hospital with regard to the treatment of chronic rheumatism is almost unique, and anybody desirous of studying the clinical aspect of chronic rheumatism could find no better field for his researches than the wards of this hospital.

Elementary Text-book of Anatomy. By Henry Edward Clark. (London: Blackie and Son. 5s.)

This little book of some 250 pages is intended chiefly for beginners or for those whose professional duties demand an elementary knowledge of anatomy. Amongst the latter class nurses and schoolmasters will find considerable assistance from the perusal of its contents. The method that the author has adopted is to divide his subject into seven sections. The first deals with histology, the second, the third, and the fourth with osteology, arthology, and myology respectively. The illustrations, which have been drawn from various sources, are numerous and well chosen, and are for the most part somewhat diagrammatic, so that the student is not confused by unnecessary detail. The illustration of the ear on the right side, which shows the external, middle, and internal portions, is one that we do not remember to have seen elsewhere: it is an exceedingly useful diagram, since it shows the position in space of the three semi-circular canals and their relationship to the cochlea and ossicles. The student of physiology as well as of anatomy should be grateful for so lucid an illustration of this difficult region. The glossary at the end of the work, giving as it does the derivation of all the anatomical names in the text, will be of use to the student of inquiring mind. For lucidity, accuracy, and interest we can recommend this little text-book.

